# Eexperimental study of interface behaviour of geopolymer concrete

**DOI:** 10.1038/s41598-025-20036-3

**Published:** 2025-09-24

**Authors:** Karim Mohsen, Ibrahim A. Yousif, Ehab F. Sadek, Khalid M. Morsy

**Affiliations:** Civil Engineering Department, Faculty of Engineering, Ain Shams University, Cairo, Egypt

**Keywords:** Geopolymer concrete (GPC), Conventional concrete (CC), Bond behavior, Flexural behavior, Fly ash, Alkali activation, Retrofitting, Mechanical properties, Engineering, Materials science

## Abstract

Flexural strengthening of reinforced concrete (RC) elements by incorporating additional layers of concrete is a widely employed technique for enhancing structural capacity. Typically, during design, the strengthened element is treated as monolithic to simplify calculations. However, this assumption often overlooks the significance of interface slip, a critical factor influencing the level of damage sustained by the strengthened element. The extent of interface slip depends on the bond strength and the surface preparation techniques used. Addressing this gap, this study undertook an experimental investigation to assess the bond strength of geopolymer concrete using manual roughening with a chisel and hammer, as well as sandblasting, in combination with epoxy-based and cement-based bonding agents. The bond strength was evaluated through slant shear and pull-off tests. Results showed that geopolymer concrete with 100% slag achieved the highest shear bond strength of 27.5 MPa in slant shear and 1.8 MPa in pull-off tests, representing an improvement of up to 25–35% compared to Portland cement concrete under similar interface conditions. Manual roughening combined with an acrylic bonding agent resulted in a 40–50% higher bond strength compared to sandblasting and epoxy bonding combinations. The findings of these tests underscore the potential of geopolymer concrete as a viable repair material for structures, owing to its commendable bond strength performance. These results contribute valuable insights into optimizing strengthening techniques for enhanced structural integrity and longevity.

## Introduction

### Environmental impact of PCC

The greatest challenge in fighting climate change comes from the production of cement which is considered the basic constituent of Portland cement concrete (PCC), which represents about 10–12% of the concrete volume and the world’s appetite for it seems insatiable. The demand for Portland cement (PC) has recently increased, resulting in a rise in its production, where the global production of PC is over 3 billion tons per year^1^ and it is predicted that PC demand will increase to be over 6 billion tons per year in the upcoming forty years.

**Fig. 1:**
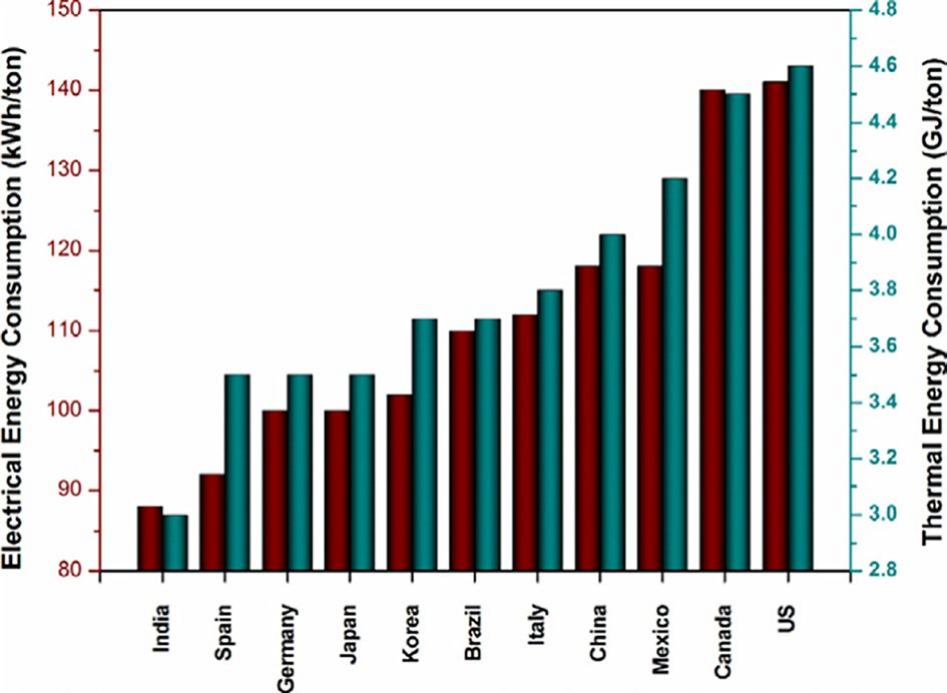
Required Electrical and thermal energy consumption for producing Portland cement for various countries^5^.

PCC remains the workhorse of the construction industry. It is manufactured by heating limestone or chalk with clay to approximately 1450 °C in a rotary kiln, producing clinker that is then ground with gypsum. This process consumes large amounts of fuel, typically coal or petroleum coke. Beyond energy use, PCC manufacture poses environmental hazards such as depletion of natural resources, dust emissions during transportation, and noise from quarrying and raw material processing. Hutchinson et al.^2^ emphasized that Portland cement production inevitably releases significant amounts of CO₂, both from the combustion of fuels needed for high kiln temperatures and from the calcination of limestone during clinker production. Moreover, Portland cement production is an energy–intensive industry where the required energy is about 50–60% f the production cost^3^. Portland cement production consumes a high amount of electrical and thermal energy, for example, to produce one ton of clinker, we need about 110–120 kWh and about 3000–6500 MJ^4^. For various countries, the average consumption of electrical and thermal energy for Portland cement production is presented in Fig. 1^5^.

### Literature review

Geopolymer concrete (GPC) is high in aluminosilicate and has characteristics such as cement. because these GP composites are unreactive in the presence of water, they require an alkaline media to work properly^6^. Furthermore, when combined with an alkaline binder, these GPs alternative binders can support load by forming a 3-D polymeric structure. The cement component reacts quickly with water because of its high natural alkaline concentration^6^. In order to form a binding with the neighboring matrix, an alkaline binder must be activated in an alkaline media. GPC offers several advantages over traditional Portland cement concrete, making it an attractive alternative for sustainable construction. It is highly durable, with excellent resistance to chemical attack, including acids, sulfates, and chlorides, making it ideal for harsh environments. GPC also exhibits superior heat and fire resistance, as it can withstand higher temperatures without significant loss of strength. Additionally, it has a lower carbon footprint because it uses industrial by-products such as fly ash or slag as binders instead of cement, reducing greenhouse gas emissions. Its early strength gain and reduced shrinkage further enhance its performance and make it suitable for various applications, including infrastructure, marine structures, and precast elements. Understanding these benefits in the context of repair applications requires examining how GPC interacts with existing Portland cement substrates, particularly in terms of interfacial bond behavior and failure mechanisms. As recently reviewed^7^, GPC typically exhibits higher bond stiffness compared to OPC due to its denser ITZ structure, but was found to lack sufficient quantitative data correlating binder composition with bond strength.

**Fig. 2:**
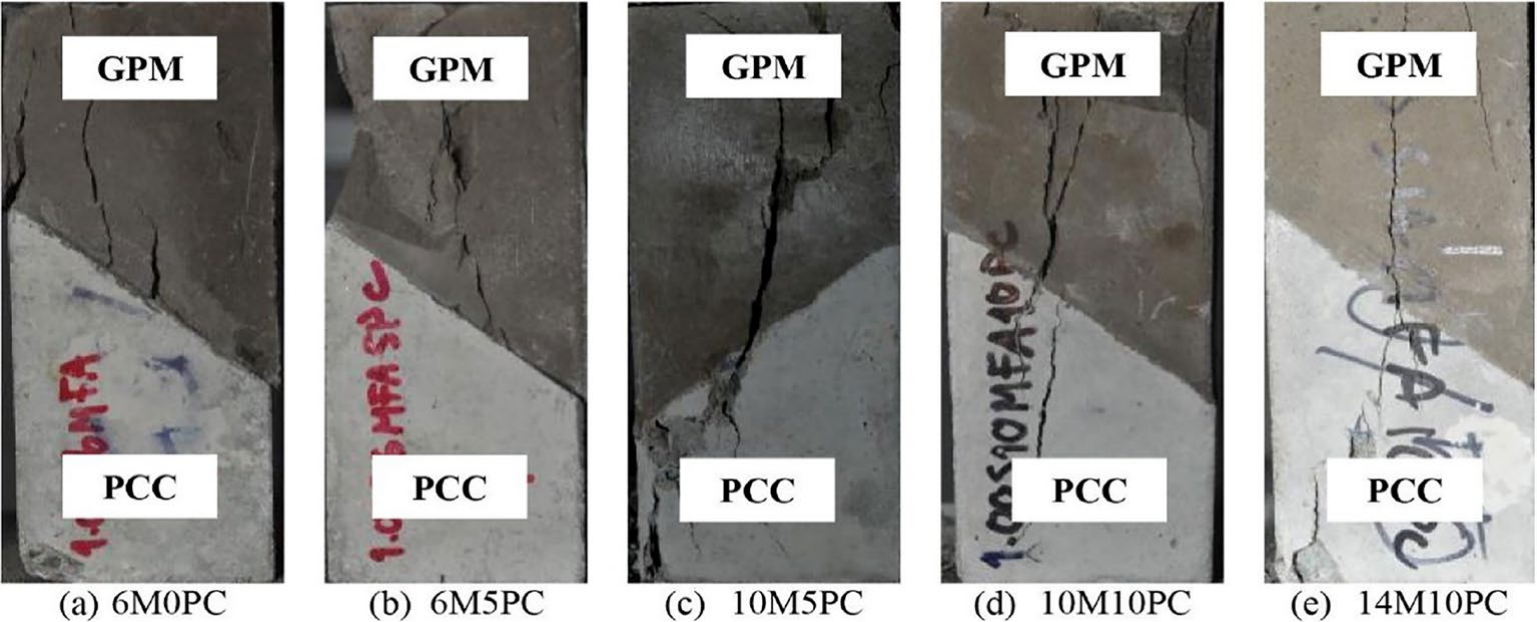
Fracture surface between PCC substrate and GPM. (Adapted from^9^.

Mix design parameters, such as binder composition and aggregate content, have been shown to influence the mechanical and interfacial performance of repair mortars. Kumar et al.^8^ reported that increasing the sand-to-binder ratio in engineered geopolymer composites enhanced bond strength with concrete substrates, highlighting the importance of tailoring mix design for repair applications. Phoongernkham et al.^9^ investigated the failure patterns of slant shear prisms and identified two failure modes, as shown in Fig. 2. The first was in Geopolymer Mortar (GPM), where cracks formed in the interface while the PCC substrate remained relatively intact. This failure mode was observed in GPMs with low NaOH concentrations without PC and low PC content (6M0PC and 6M5PC mixes). The slant shear bond prisms failed in monolithic mode for other mixes with relatively high strengths, high NaOH, and high PC, such as 10M10PC and 14M10PC. Cracks appeared in both the GPM and PCC substrate sections. This clearly demonstrated GPMs’ superior crack resistance and improved bonding between the two surfaces.

**Fig. 3:**
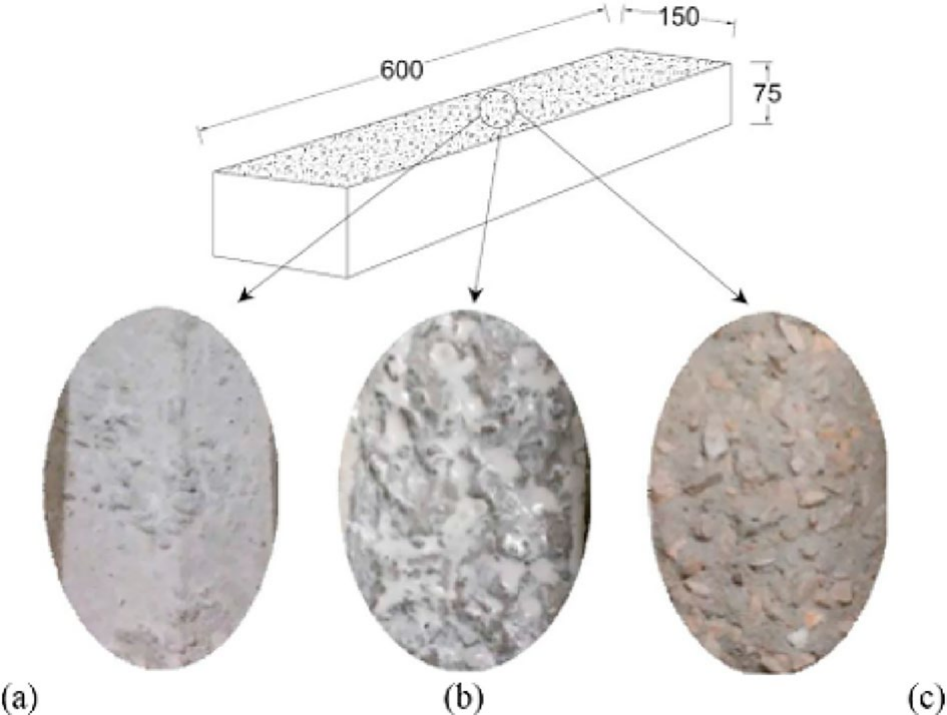
CC substrate surface before placing the GPC: (**a**) no treatment, (**b**) concrete bonding adhesive, and (**c**) sandblasting. (Adapted from^10^.

Gomaa et al.^10^ tested the binding strength of five different mixes of fly ash-based GPC as a repair layer for PCC substrates, with the sole variable being the source of the fly ash. In each 150 × 150 × 600 mm beam mould, a 75 mm high layer of PCC was put. Then, on each CC surface, either no surface treatment (Fig. 3-a), a concrete bonding glue (Fig. 3-b), or sandblasting was used (Fig. 3-c). The bonding agent was supplied by QUIKRETE and meets ASTM C1059-13 type I and II criteria. Following that, a coating of GPC mixture was applied to the PCC surface of each repaired specimen, and the mended specimens were cured. Specimens repaired with PCC were also made. For comparison, full-depth PCC and GPC beams with no cold joints were also produced.

**Fig. 4:**
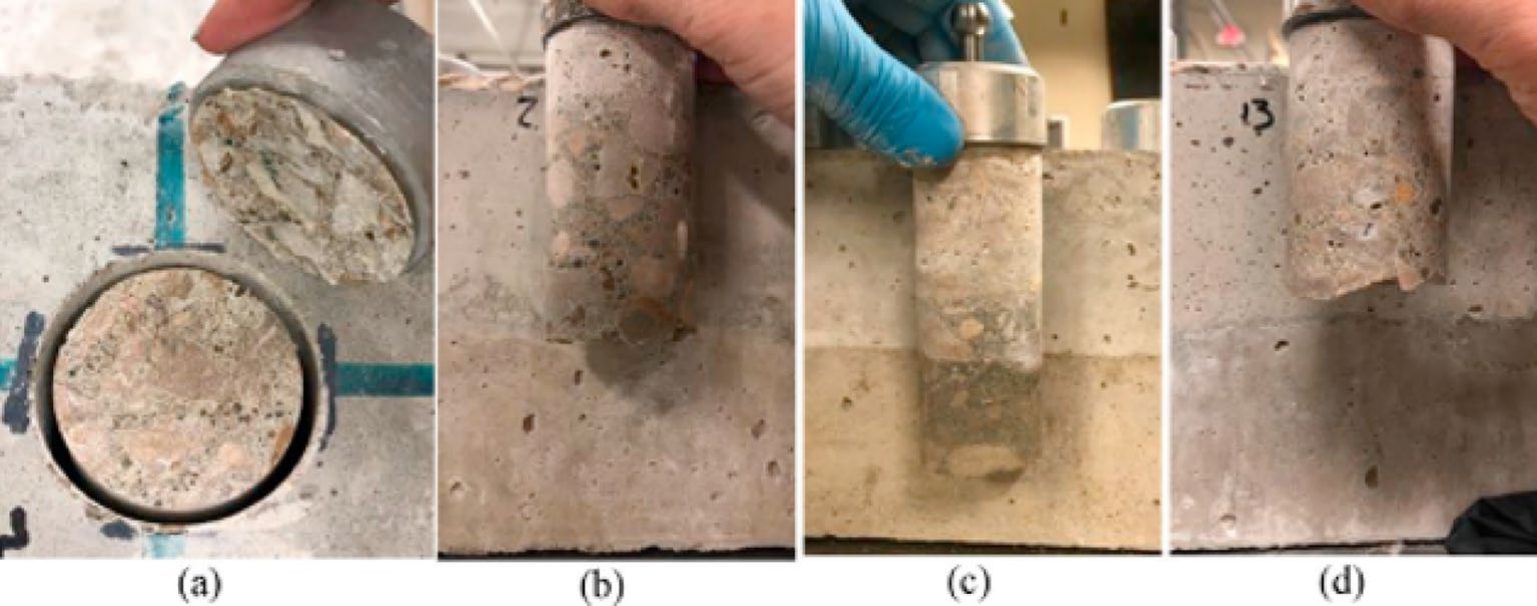
Failure of the pull-off specimens in. (Adapted from^10^.

Gomaa et al.^10^ found out the failure at the interface surface between the aluminum disc and the repair materials revealed poor epoxy, and the test was deemed a failure (Fig. 4-a). The surface treatment influenced the failure mode of the restored beams for the remaining beams. Approximately 90% of the restored beams with no surface treatment failed at the interface surface between the repair and substrate materials (Fig. 4-b), indicating weak bond strength. The repair material failed 10% of the remaining no-surface-treatment specimens (Fig. 4-d). Furthermore, within the PCC substrate material, 100% of the sandblasted beams failed (Fig. 4-c), demonstrating a good binding strength.

**Fig. 5:**
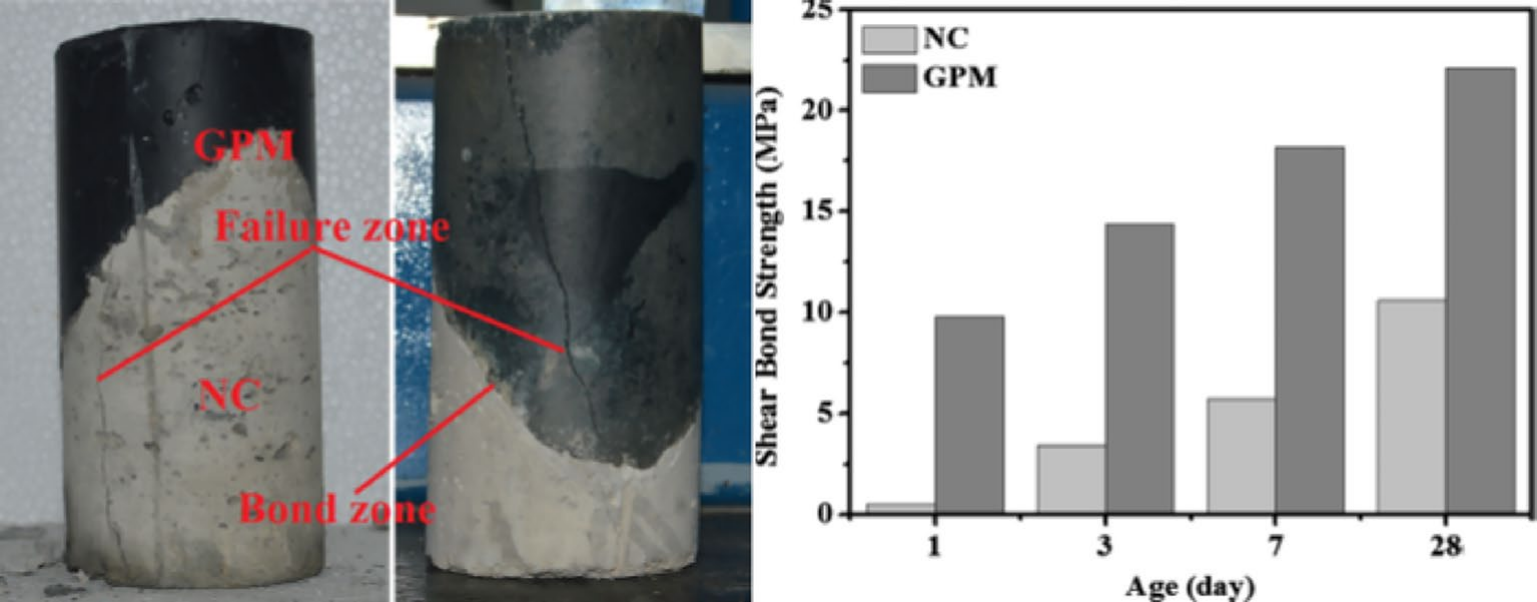
Percent of modes of failure of the test specimens (Adapted from ^11^)

**Fig. 6:**
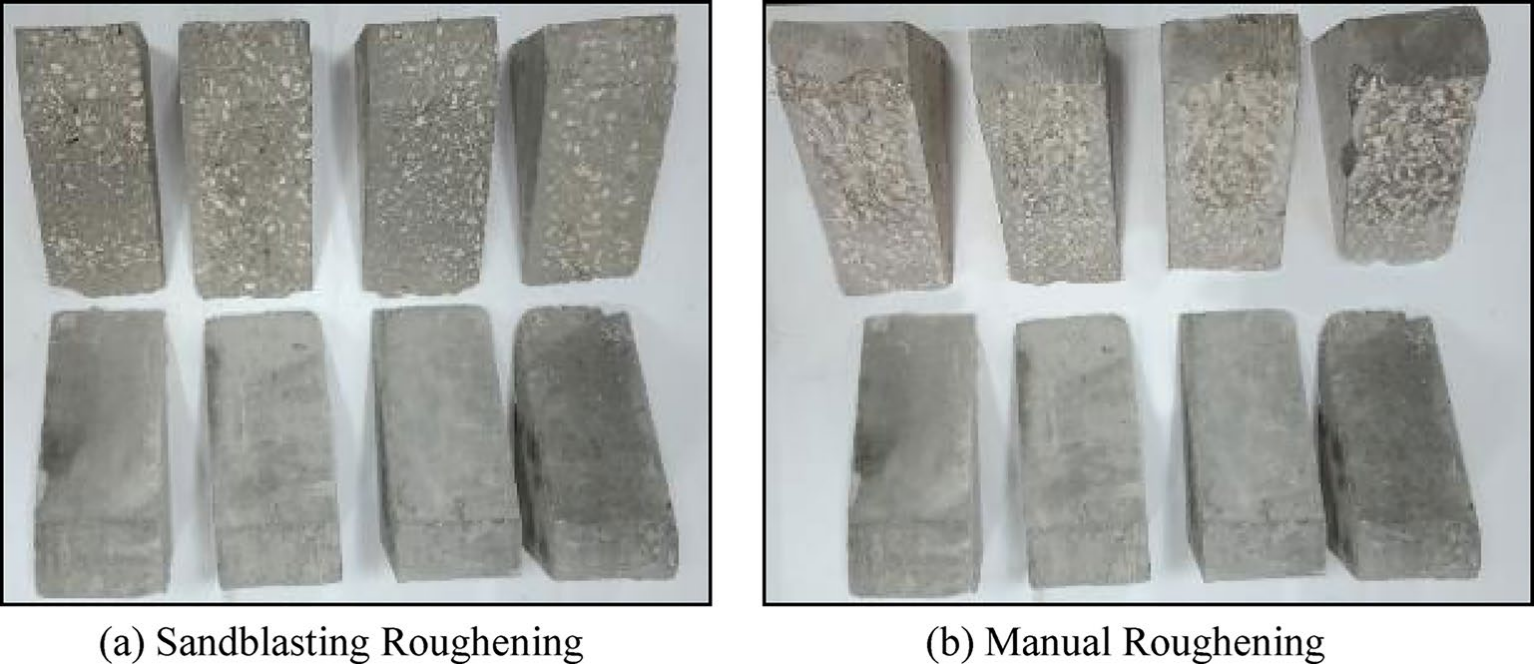
Percent of modes of failure of the test specimens. (Adapted from^11^.

Ghasan et al.^11^ used a slant shear bond test to measure the bond strength between OPC (NC) and GPM. The specimens are cylinder slant shear specimens (100 mm x 200 mm) with the contact line at 30^o^. The binding strength was measured after 1, 3, 7, and 28 days of cure at room temperature. The slant shear test is the most commonly used for determining the bonding of repair materials to concrete. Figure 5 compares the binding strength of GPMs to that of OPC mortar. When compared to OPC, the bond strength of GPM produced with 5% MK, 1.16 SiO2:Na2O, and 8% Na_2_O: dry binder was maximum at 9.9 MPa and 22.4 MPa in the early (1 day) and late (28 days), respectively. Figure 6 also depicts a typical bond breakdown in a slant shear sample, where the bonding surface was discovered to be intact. The fissures cut through the NC substrate and the GPM interface. Furthermore, no considerable gap existed between the two bonding surfaces, as corroborated by other reports^12^.

Numerous studies have been conducted in recent years to improve the strength of geopolymer products, investigate the fresh and hardened characteristics of geopolymer, and comprehend the mechanism of geopolymerization. When choosing geopolymers as repair materials, the bond strength between the substrate concrete and the repair material is critical. Because most of these tests were conducted on paste and mortar rather than concrete, further research is needed before GPC may be used as a retrofitting material. In this context, the novelty of the present study lies in evaluating concrete-to-concrete bond performance using GPC repair systems, rather than focusing solely on paste or mortar. Moreover, this research investigates the combined influence of surface roughening techniques, bonding agents, and binder composition on bond strength—an integrated approach that has not been comprehensively addressed in previous studies. By incorporating both quantitative surface roughness characterization (SEM and 3D profiling) and bond strength testing (slant shear and pull-off), this work provides a more holistic understanding of interfacial mechanisms relevant to structural retrofitting.

## Methodology

The primary objective of this experimental study is to investigate the bond behavior between GPC and substrate Portland cement concrete (PCC) for various levels of constituent materials and surface preparation techniques using slant shear and pull-off tests. Two bonding agents were used, one with epoxy-based material and the other with cement-based material. Also, two roughening techniques were adopted manual roughening using a chisel and hammer, and sandblasting roughening.

The results of the experiments are analyzed to determine the effects of material composition and surface preparation on the bond strength between GPC and PCC. This methodology provides a comprehensive approach to understanding the factors influencing bond behavior, offering insights for enhancing the performance of repair and composite construction applications.

## Experimental program

### Material characterization

GPC was produced using pure GGBFS and FA due to their cementitious and pozzolanic properties. The GGBFS (off-white in appearance) in particular was selected for its higher calcium content, which promotes rapid geopolymerization and contributes to early strength development, making it especially suitable for repair applications. Sodium hydroxide solution (NaOH) (60.25% Na_2_O, and 39.25% H_2_O) and sodium silicate solution (11.98% Na_2_O, 31.00% SiO_2_, and 57.00% H_2_O) were used as liquid activators. PCC was produced using cement as the main binder while the activator was only by using water to form hydration products. Table 1 summarizes the composition of GGBFS, FA and Portland cement using the X-ray fluorescence (XRF) test. The locally available natural sand with a nominal maximum particle size of 5 mm and the crushed limestone with a nominal maximum size of 10 mm were used for fine aggregate and coarse aggregate, respectively. The specific gravity was 2.62 for fine aggregate while specific gravity and water absorption were 2.60 and 0.98%, respectively, for coarse aggregate. Table 2 summarizes the physical and chemical properties of used fine and coarse aggregate which met the continuous standard recommendations in ASTM C33.

**Table 1:** Chemical composition of GBFS, FA and PC.

Materials	SiO_2_	Al_2_O_3_	CaO	MgO	Fe_2_O_3_	MnO	TiO_2_
GGBFS	35.10	16.90	37.50	7.85	1.30	0.52	0.23
FA	53.50	27.80	1.90	0.90	11.20	0.20	3.20
PC	19.02	4.34	63.25	0.77	3.45	0.26	0.28

GGBFS: Ground Granulated Blast Furnace Slag.

FA: Fly Ash.

PC: Portland cement.

**Table 2:** Chemical and physical properties of fine and coarse aggregate.

Materials	NMS (mm)	Unit weight (kg/m^3^)	Specific gravity	Water absorption (%)	Fine material content (%)	Chloride content (%)	Sulphate content (%)
Fine Aggregate	---	1590	2.62	--	2.40	0.0285	0.1453
Coarse Aggregate	10	1550	2.60	1.50	0.46	0.0137	0.2957

### Mixtures proportions

Two types of industrial waste materials (FA and GGBFS) were used to prepare the GPC mix design. The water-to-FA or GGBFS, alkaline-to-FA or GGBFS, SS/SH, and all other factors affecting the mix design of GPC were selected based on a comprehensive trial mixture^13^. All aggregates were batched in a saturated surface dry state. The CC mixture’s proportions were selected to achieve a 28-day compressive strength comparable to that of the geopolymer mixture. Table 3 summarizes the mix proportions of two GPC mixes and one Portland cement concrete mix, which were prepared during this study.

**Table 3:** Mix proportions of GPC and PCC (kg/m^3^).

Mixes	GGBFS	FA	PC	Na_2_SiO_3_	NaOH	Water	W/B	F.A	C.A	Admixture (%)
SGC	450	0	0	131	41	112	0.45	547	1093	0
FSGC	270	180	0	131	41	112	0.45	547	1093	0
PCC	0	0	450	0	0	198	0.45	703	1055	1

SGC: Slag-based GPC.

FSGC: (Fly ash + slag) based GPC.

PCC: Portland cement concrete.

W/B: Water to binder ratio.

F.A: Fine Aggregate.

C.A: Coarse Aggregate.

Admixture: water-reducing agent and super- plasticizer.

### Concrete manufacturing

The procedure for mixing GPC implemented in this study started with mixing the dry materials (GGBFS or FA and aggregates) in a pan mixer for 1 min. After that, the alkaline activator was added to the dry mix and stirred for about 4 min, or until the mixture became homogeneous. Before adding the alkaline activator to the dry mix, the SH pellets, SS solution, and water were mixed for about 1 h. After 24 h, the specimens were removed from the molds and cured in the laboratory at an ambient temperature of 25 ± 2 °C for the GPC mixes and in water curing tanks for the PCC mixes until testing.

### Test matrix

To investigate the effect of the different parameters affecting bond strength, such as surface roughness and bonding agent, a test matrix has been planned for both the slant shear and pull-off tests. The test matrix is summarized in Table 4.

**Table 4:** Slant shear and pull of test matrix.

Specimen designation	Binder of repair concrete	Surface roughness	Bonding agent
SSA	Slag	Sandblasting	Acrylic
SSE	Slag	Sandblasting	Epoxy
SMA	Slag	Manual	Acrylic
SME	Slag	Manual	Epoxy
FSSA	Fly Ash + Slag	Sandblasting	Acrylic
FSSE	Fly Ash + Slag	Sandblasting	Epoxy
FSMA	Fly Ash + Slag	Manual	Acrylic
FSME	Fly Ash + Slag	Manual	Epoxy
SM	Slag	Manual	--
CM	Cement	Manual	--
CMA	Cement	Manual	Acrylic

## Results and discussion

### Surface preparation techniques

In repairing concrete structures, the preparation of the substrate surface is considered one of the most important stages. This is to ensure that the substrate surface will be bonded mechanically with the new repair material. During this phase, the substrate concrete surface was prepared in two steps. The first step is the roughening of the substrate concrete surface, and the second step is the application of bonding agents at the substrate concrete surface to ensure the development of a proper bond with the new concrete. The objective of having different surface preparation techniques was to evaluate and discuss the influence of each parameter on the quality of bonding between the geopolymer and concrete substrates.

#### Surface roughness

**Fig. 7:**
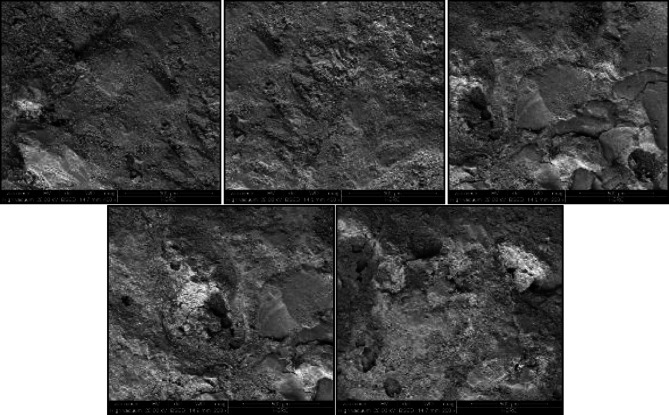
Two dimensional images for sandblasting roughening technique samples.

The samples of PCC substrate were cured for 28 days to ensure that the concrete gained sufficient strength. Then, roughening preparations were carried out on the surface of the concrete. Two types of surface textures were adopted and used in this study, which were manual roughening (M) using a chisel and hammer, and sandblasting roughening (S). Both roughening techniques were applied until particles of coarse aggregates appeared on the surface of the concrete. This zigzag in the surface of concrete, due to the appearance of parts of coarse aggregates, would ensure the development of a proper bond with the new concrete. Figure 7 shows the concrete surface before and after roughening techniques.

**Fig. 8:**
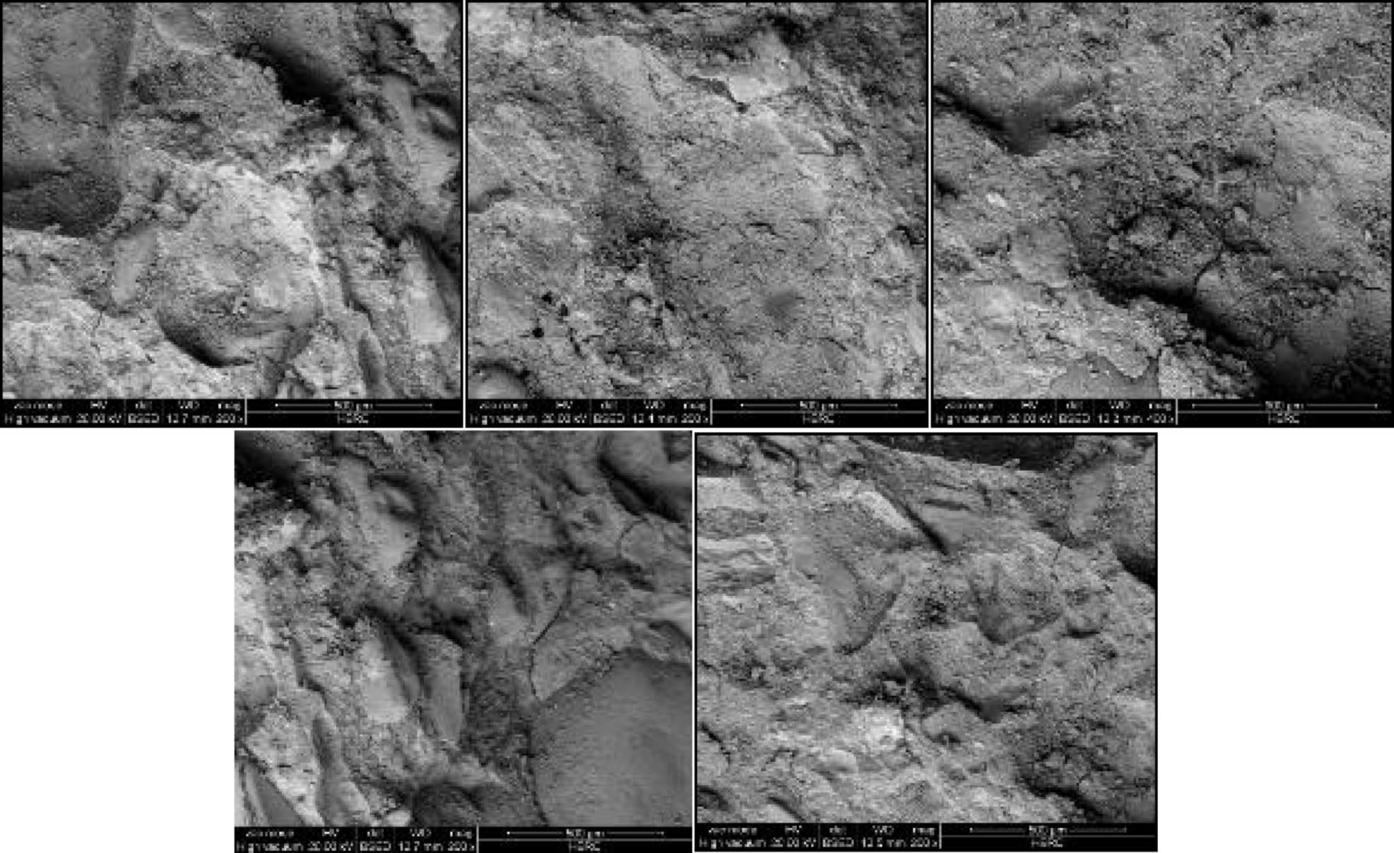
Two-dimensional images for manual roughening technique samples.

The surface topography of both techniques was measured to detect the more roughened surface. The fractured surface for selected samples was observed using a scanning electron microscope (SEM) (Model xT810 Inspect S/2007 electron microscope) operating at an accelerating voltage of 5 kV. Two-dimensional images were taken from the samples extracted from each roughened cube. Then the two-dimensional images were transformed using software attached to the scanning electron microscope device into three-dimensional images to detect the elevation of surface texture. Figure 5 presents the two-dimensional images recorded for sandblasting roughening technique samples, while Fig. 8 presents the two-dimensional images recorded for manual roughening technique samples.

**Fig. 9:**
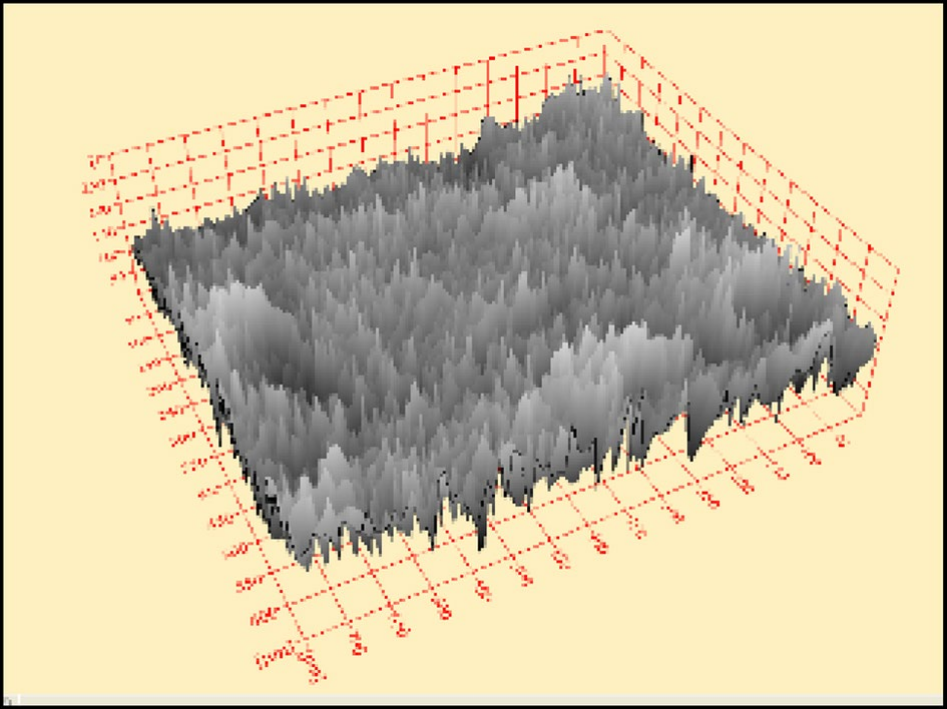
Three-dimensional image for sandblasting roughening technique sample.

**Fig. 10:**
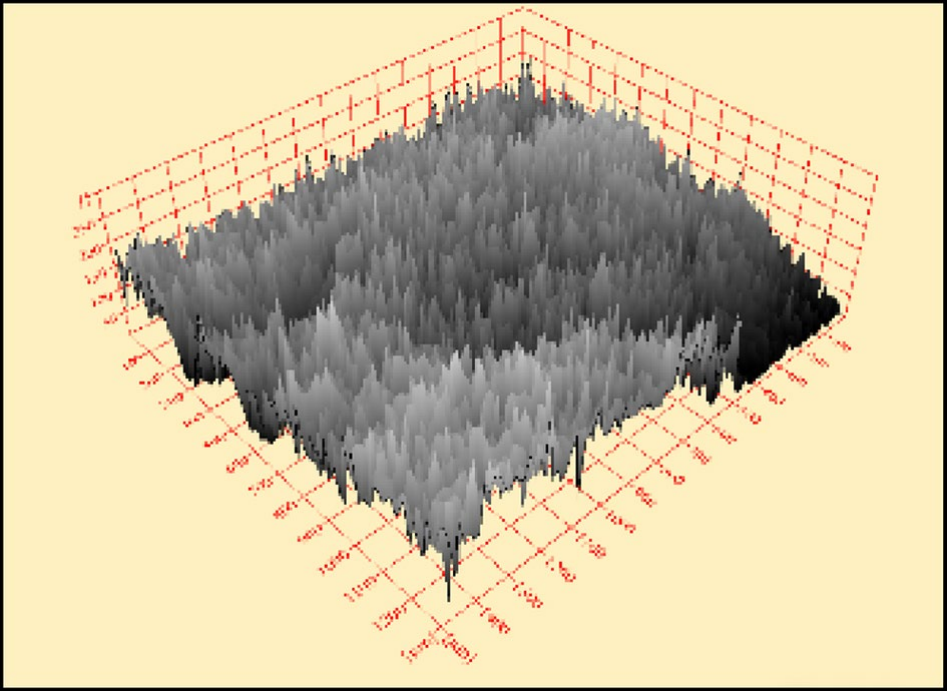
Three-dimensional image for manual roughening technique sample.

By using software (Scandium-3D surface) attached to SEM, the two-dimensional images for both roughening techniques recorded from SEM were transformed into three-dimensional images. Figure 9 represents the transformed two-dimensional image for a single sample taken from a sandblasting-roughened cube. while Fig. 10 represents the transformed two-dimensional image for a single sample taken from a manually roughened cube.

**Fig. 11:**
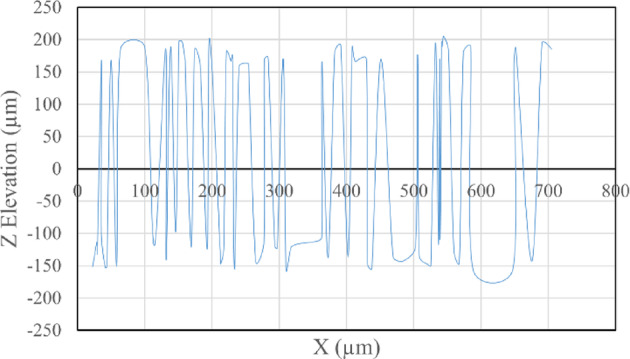
X location plotted against Z elevation for sandblasting roughening technique.

**Fig. 12:**
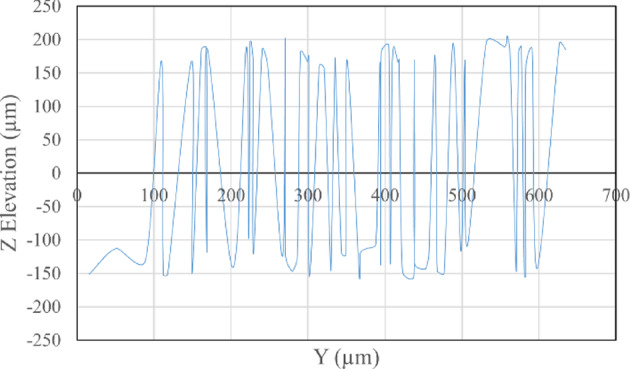
Y location plotted against Z elevation for sandblasting roughening technique.

**Fig. 13:**
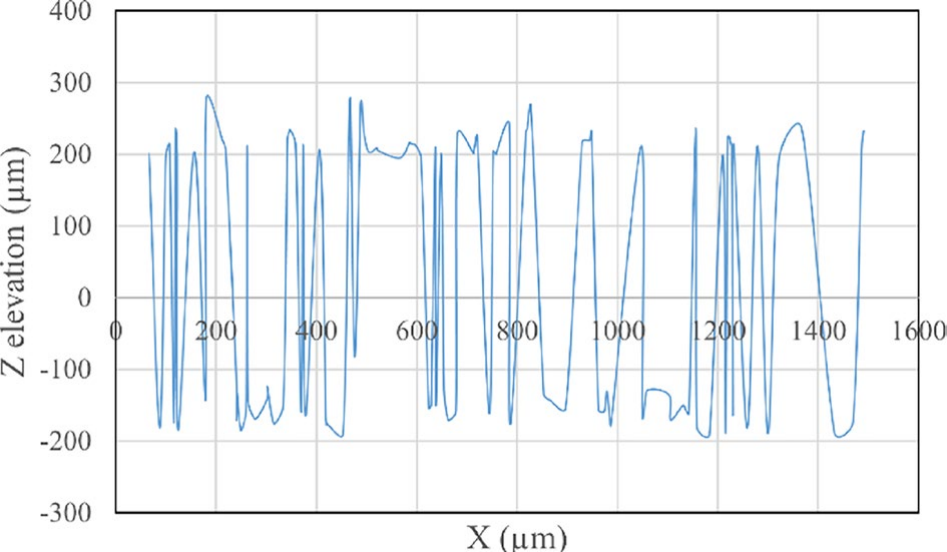
X location plotted against Z elevation for manual roughening technique.

**Fig. 14:**
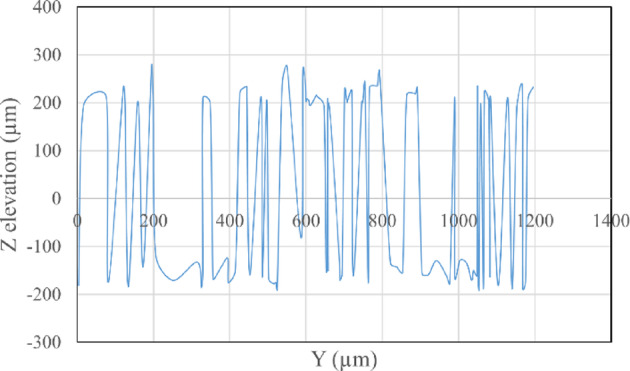
Y location plotted against Z elevation for manual roughening technique.

The average Z elevation for the five samples taken from each roughened cube was recorded in (µm), and the X and Y location of chosen points were plotted against Z elevation for each roughening technique to detect which roughening technique has the highest peaks and falls. Figures 11 and 12 present the X and Y location of chosen points plotted against Z elevation for the sandblasting roughening technique, while Figs. 13 and 14 present the X and Y location of chosen points plotted against Z elevation for the manual roughening technique.

The surface texture of the sandblasting roughening ranges from a minimum fall of 155.38 μm to a maximum peak of 204.89 μm with an average coefficient of variance of 17.76% for all samples. The surface texture of the manual roughening ranges from a minimum fall of 190.73 μm to a maximum peak of 275.19 μm with an average coefficient of variance of 28.30% for all five samples. From Figs. 11, 12 and 13, and 14, it can be concluded that the manual roughening technique has a higher absolute difference between falls and peaks than the sandblasting roughening technique. This greater difference between peaks and falls indicates that the manual roughening provides a more irregular surface profile, which enhances mechanical interlocking and frictional resistance between the PCC substrate and the geopolymer repair layer, thereby improving bond strength. Both roughening techniques were utilized in the preparation of slant shear and pull-off specimens to detect which roughening technique would give higher bond strength between the PCC substrate and GPC.

To provide a quantitative comparison of surface textures, the arithmetic mean roughness (Ra) and mean peak-to-valley height (Rz) were calculated from the SEM-derived 3D surface profiles. The sandblasting roughening achieved Ra = 150–250 μm and Rz = 600–900 μm, while the manual roughening technique achieved Ra = 300–500 μm and Rz = 1200–1800 μm. These parameters confirm that the manual roughening technique generated a significantly rougher and more irregular surface, which is expected to enhance mechanical interlocking and bond strength.

#### Bonding agents

The concrete bonding agents are natural or chemical materials utilized to join the old and new concrete surfaces. This agent can also be utilized to join the successive concrete layers. This bonding agent helps to allow different concrete finishes to behave as a single unit. Two types of bonding agents were selected in this study: an acrylic-based bonding agent and an epoxy-based bonding agent. Acrylic bonding agents are commonly used in repair applications due to their good workability, ease of application, and ability to improve adhesion with cementitious substrates. Epoxy bonding agents, on the other hand, are widely employed in structural retrofitting because of their superior adhesive strength and durability. The choice of epoxy is further supported by previous research demonstrating the successful use of epoxy-based systems for bonding geopolymer composites with reinforcement, including CFRP and FRP strengthening applications^14,15^.

### Slant shear test

The slant shear test was performed according to the ASTM C882 standard^16^ to assess the interfacial shear strength between the GPC as a repair concrete and the PCC as a substrate concrete. Table 5 summarizes the results of the slant shear test.

It was found that shear bond strength after 28 days in the case of using GPC with 100% slag is higher than shear bond strength in the case of using GPC with 70% slag and 30% fly ash after 28 days. Also, similar trends in shear bond strength development were observed at the ages of 3 and 7 days.

In the case of using a combination of latex bonding agent and manual roughening technique, shear bond strength was higher compared to other combinations of bonding agents and roughening techniques, with both types of GPC used, as can be seen in Table 5. The specimens repaired with 100% slag GPC without bonding agent gave higher shear bond strength than specimens repaired with 100% slag GPC and with epoxy bonding agent, but lower shear bond strength than specimens repaired with 100% slag GPC and with latex bonding agent.

For control specimens without bonding agents, it was observed that shear bond strength is higher than all other specimens’ combinations except for specimens repaired with 100% slag GPC and with a combination of latex bonding agent and manual roughening technique. Usually, specimens repaired with epoxy bonding agent showed lower shear bond strength than specimens repaired with latex bonding agent. Also, specimens repaired with the sandblasting roughening technique showed lower shear bond strength than specimens repaired with the manual roughening technique.

**Fig. 15:**
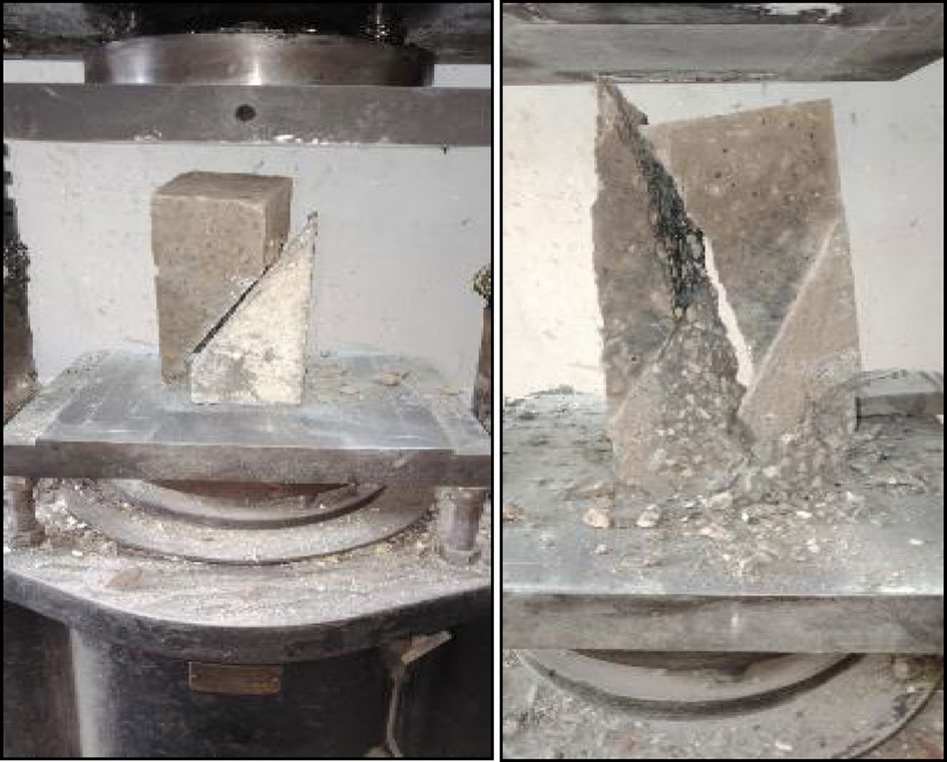
Failure modes for slant shear test.

Two modes of failure were observed during the test: the first mode was sliding the two halves of the slant shear prism on each other, and the second mode of failure was splitting cracks that appeared either on the whole prism at once, crossing the bonded surface, or only at the PCC substrate. The only two combinations that gave a splitting mode of failure were detected when using GPC with 100% slag, acrylic bonding agent with manual roughening technique, and control specimens Fig. 15 shows the two modes of failure detected during the test.

Ghasan^17^ concluded that the higher the ratio between GGBFS and FA as a binder for GPM, the higher the shear bond strength. Also, the higher shear bond strength of GPM was detected but on mortars, not concrete^11^.

**Table 5:** Slant shear test results at 28 days.

Specimen designation	Bond strength (MPa)	Mode of failure
SSA	14.30 ± 0.97	Sliding
SSE	9.00 ± 1.05	Sliding
SMA	27.50 ± 2.41*	Splitting
SME	12.40 ± 1.05*	Splitting
FSSA	8.54 ± 0.75	Sliding
FSSE	7.54 ± 0.75	Sliding
FSMA	12.85 ± 1.71	Sliding
FSME	8.17 ± 1.19	Sliding
SM	22.85 ± 1.80*	Splitting
CM	21.40 ± 1.75*	Splitting
CMA	23.50 ± 2.00*	Splitting

* Shear stress because the mode of failure is splitting not sliding.

** Bond strength values represent mean ± standard deviation for three specimens.

Failure modes were classified based on visual inspection of fracture surfaces and crack propagation patterns recorded during testing.

– **Sliding failure** was identified when separation occurred along the interface plane with minimal crack penetration into the substrate.
– **Splitting failure** was characterized by through cracks extending across the bonded section, often accompanied by a sudden load drop.

#### Slant shear test parametric study

This study was done to evaluate the effect of each parameter included in the slant shear test on the shear bond strength between GPC as a repair layer and Portland cement concrete as a substrate layer.

##### Effect of bonding agent

**Fig. 16:**
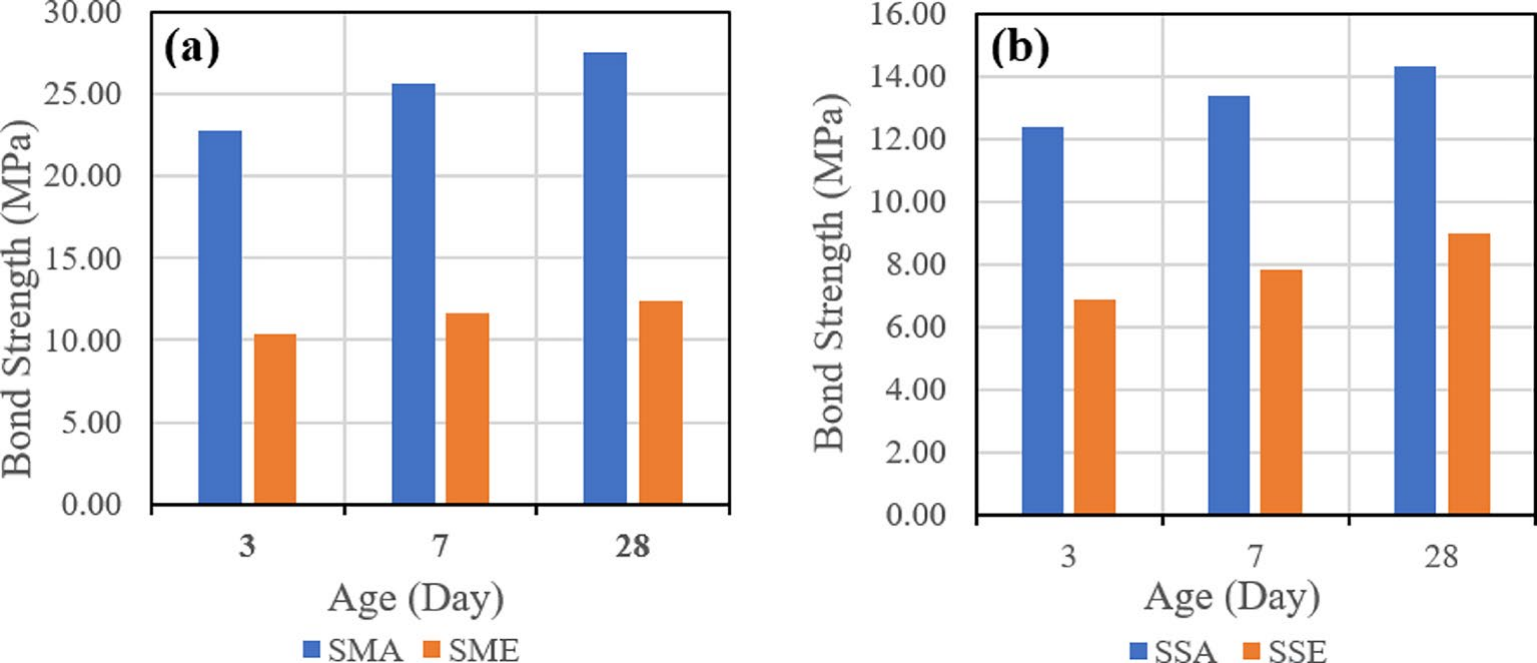
Effect of bonding agent on slant shear bond strength for slag-based geopolymer concrete (SGC) using (**a**) manual roughening and (**b**) sandblasting roughening at 28 days.

The first evaluated parameter is the effect of the bonding agent on the shear bond strength. Figure 16 indicates the effect of used bonding agents when the same mix is used, which is (SGC), and the same roughening technique, which is the manual roughening in (a) and sandblasting roughening in (b).

**Fig. 17:**
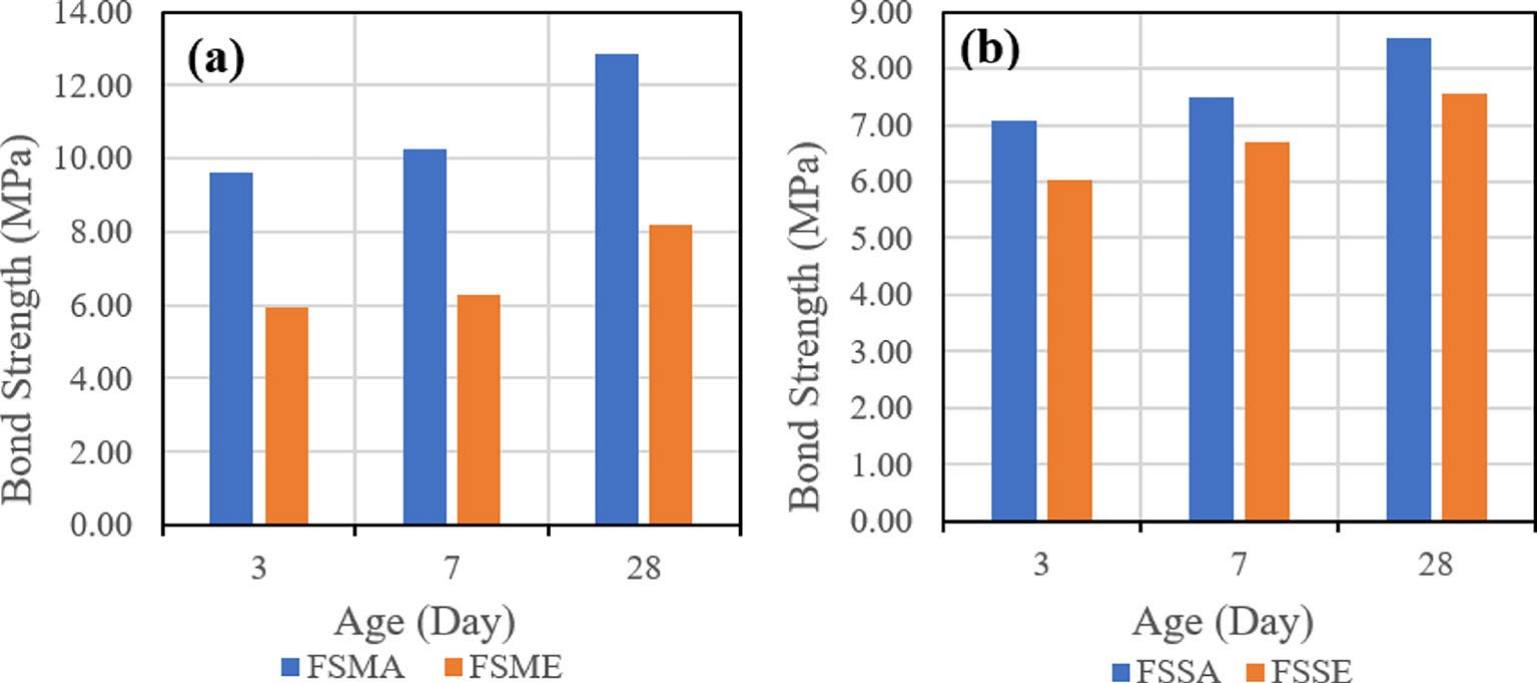
Effect of bonding agent on slant shear bond strength for fly ash–slag–based geopolymer concrete (FSGC) using (**a**) manual roughening and (**b**) sandblasting roughening at 28 days.

Figure 17 also represents the effect of used bonding agents when the same mix is used, which is (FSGC), and the same roughening technique, which is the manual roughening in (a) and sandblasting roughening in (b).

It was observed that in the two mixes, SGC and FSGC, the inclusion of acrylic bonding agent gave higher shear bond strength than epoxy-based bonding agent. Also, the influence of the bonding agent is clear in the case of the SGC mix, as the difference between the two roughening techniques is higher than FSGC.

##### Effect of surface roughness

**Fig. 18:**
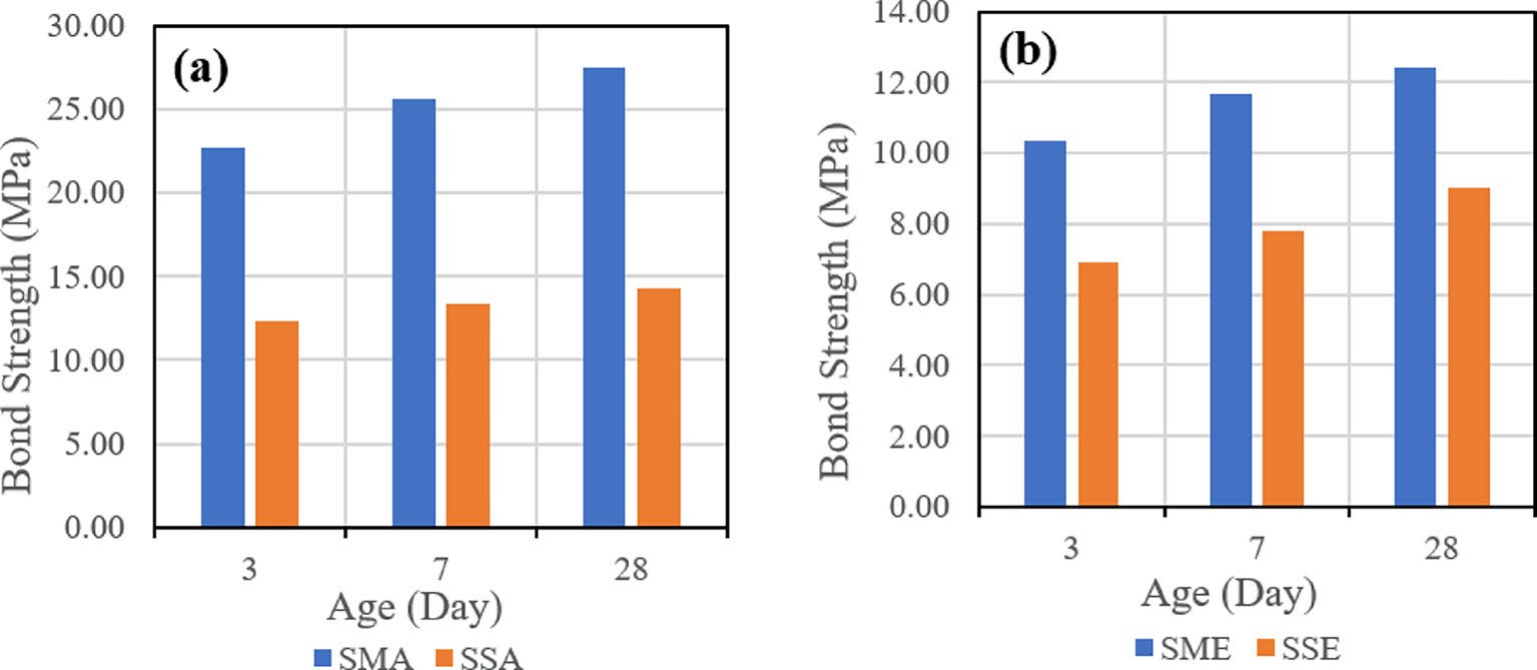
Effect of surface roughness (manual vs. sandblasting) on slant shear bond strength for slag-based geopolymer concrete (SGC) using (**a**) acrylic bonding agent and (**b**) epoxy bonding agent at 28 days.

The second evaluated parameter is the effect of surface roughness on the shear bond strength. Figure 18 indicates the effect of the two types of surface roughness used when the same mix is used, which is (SGC) and the same bonding agent, which is the acrylic bonding agent in (a) and the epoxy bonding agent in (b).

**Fig. 19:**
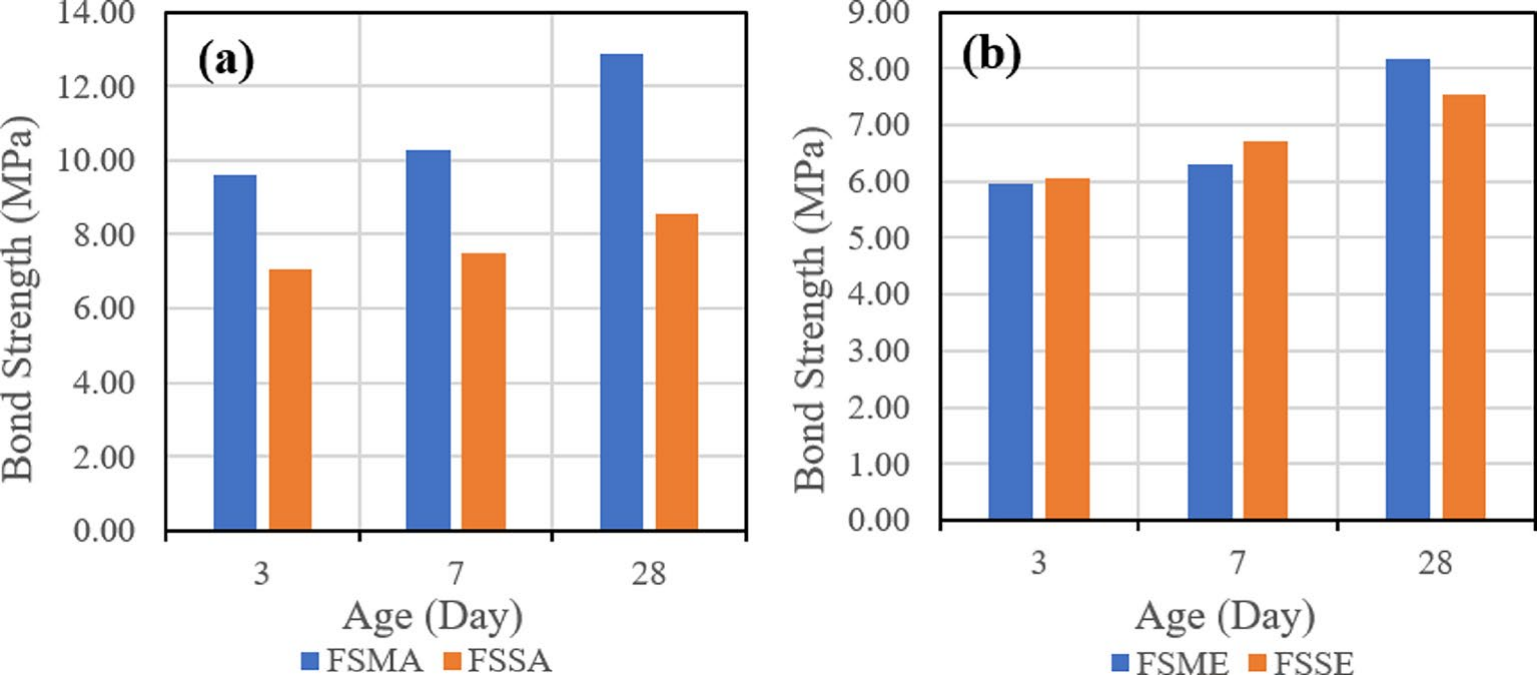
Effect of surface roughness (manual vs. sandblasting) on slant shear bond strength for fly ash–slag–based geopolymer concrete (FSGC) using (**a**) acrylic bonding agent and (**b**) epoxy bonding agent at 28 days.

Figure 19 also represents the effect of used surface roughness when the same mix is used, which is (FSGC), and the same bonding agent, which is the acrylic bonding agent in (a) and the epoxy bonding agent in (b).

It was observed that in the two mixes, SGC and FSGC, the usage of the manual roughening technique gave higher shear bond strength between GPC and PCC than the sandblasting roughening technique.

##### Effect of binder

**Fig. 20:**
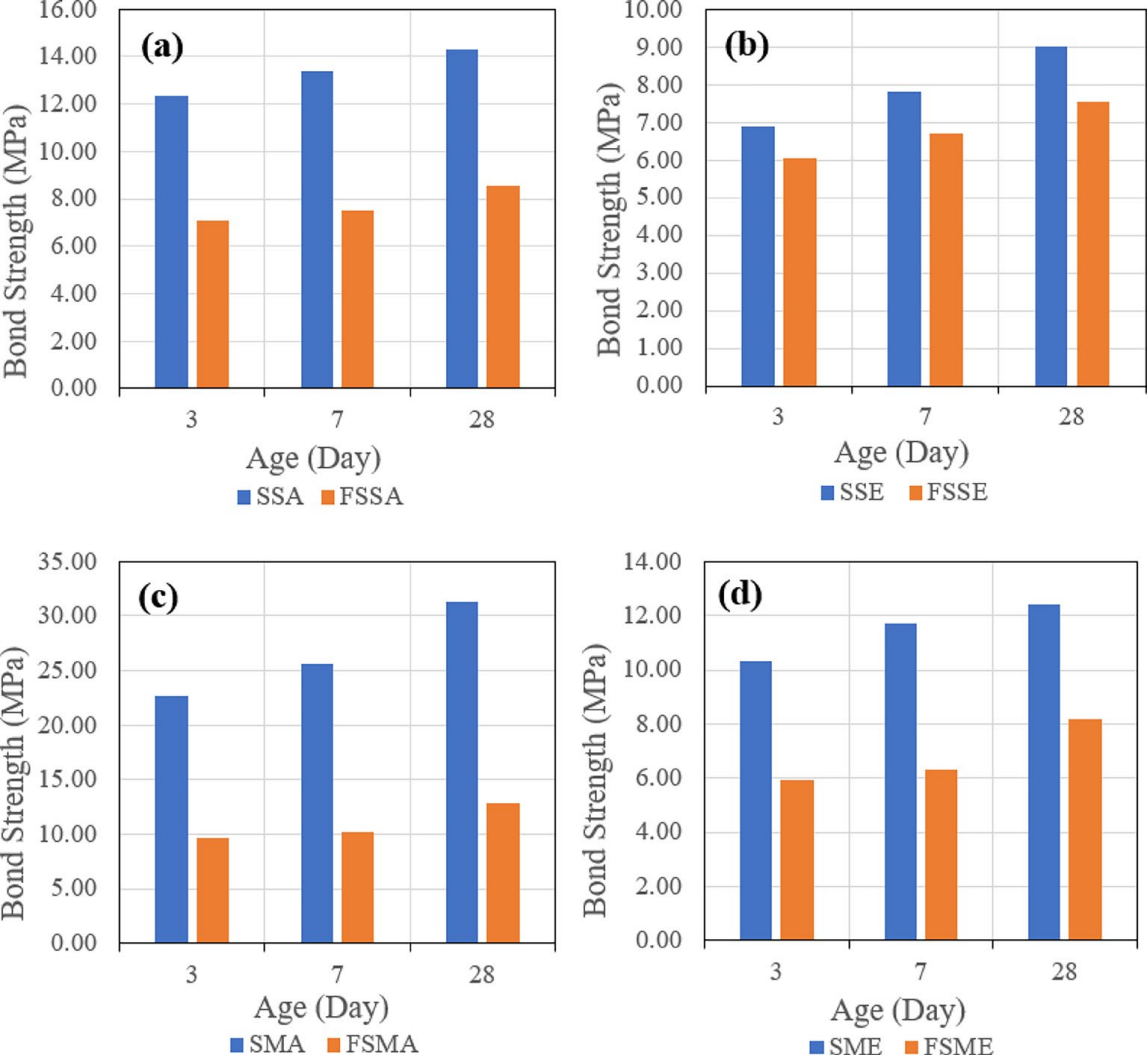
Effect of binder type (slag-based vs. fly ash–slag–based geopolymer concrete) on slant shear bond strength under combined conditions: (**a**) sandblasting + acrylic bonding agent, (**b**) sandblasting + epoxy bonding agent, (**c**) manual + acrylic bonding agent, and (**d**) manual + epoxy bonding agent at 28 days.

The third evaluated parameter is the effect of different binders on the shear bond strength. Figure 20 indicates the effect of the two types of binders when the same roughening technique and bonding agent are used, which might be one of four combinations, which are Sandblasting + Acrylic (a), Sandblasting + Epoxy (b), Manual + Acrylic (c), and Manual + Epoxy (d).

It was observed that in the four combinations, the usage of the mix (SGC), which contains 100% slag, gave higher shear bond strength.

### Pull off test

A pull-off test was performed according to the ASTM D7234 standard^18^ to assess the interfacial shear strength between the GPC as a repair concrete and the PCC as a substrate concrete. Table 6 summarizes the results of the pull off test.

Test results were consistent with the test results of the slant shear test, as it was found that shear bond strength in the case of using GPC with 100% slag is higher than shear bond strength in the case of using GPC with 70% slag and 30% fly ash after 28 days. Also, similar trends in shear bond strength development were also observed at ages of 3 and 7 days.

In case of using a combination of latex bonding agent and manual roughening technique, shear bond strength was higher compared to other combinations of bonding agents and roughening techniques with both types of GPC used. The specimens repaired with 100% slag GPC without bonding agent gave higher shear bond strength than specimens repaired with 100% slag GPC and with epoxy bonding agent but lower shear bond strength than specimens repaired with 100% slag GPC and with latex bonding agent.

For control specimens without bonding agents, it was observed that shear bond strength is higher than all other specimens’ combinations except for specimens repaired with 100% slag GPC and with a combination of latex bonding agent and manual roughening technique. Usually, specimens repaired with epoxy bonding agent showed lower shear bond strength than specimens repaired with latex bonding agent. Also, specimens repaired with the sandblasting roughening technique showed lower shear bond strength than specimens repaired with the manual roughening technique.

**Fig. 21:**
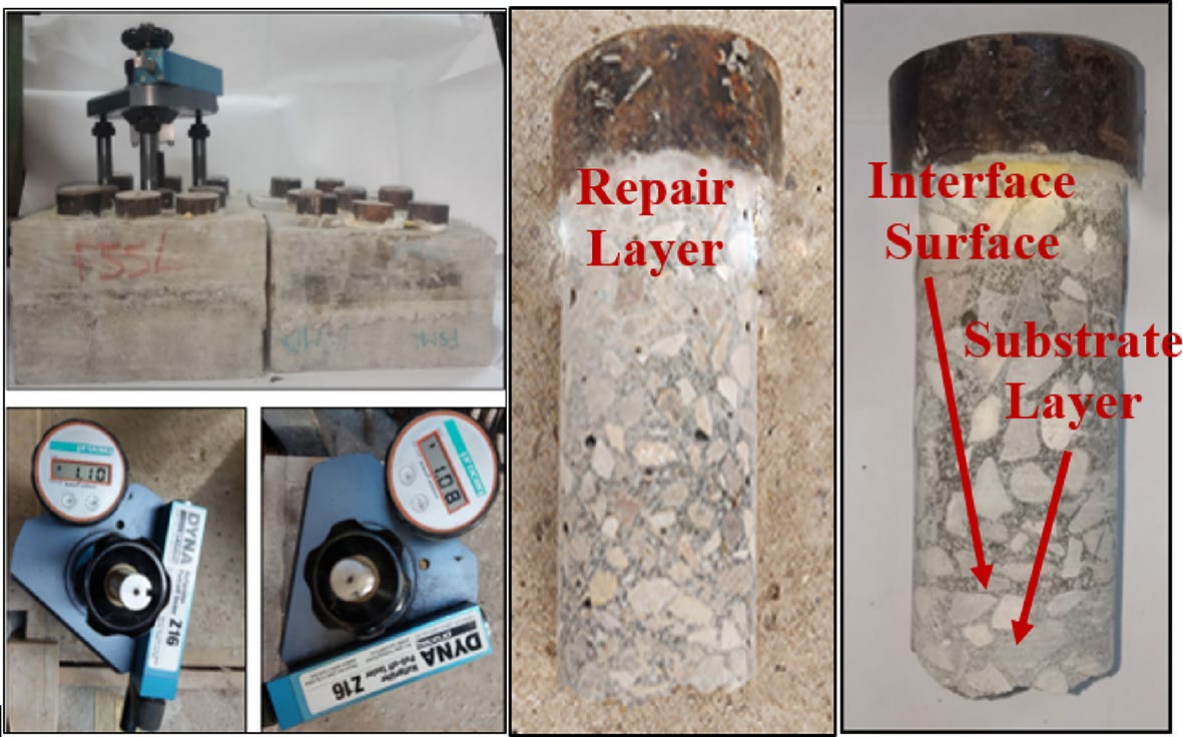
Pull off test setup and modes of failure.

Two failure locations were observed during the test; the first location was at the interface between the repair layer and substrate layer. And the second location was at the substrate layer. The only two combinations that gave a failure location at the substrate layer were detected when using GPC with 100% slag, acrylic bonding agent with manual roughening technique, and control specimens. Figure 21 shows the two modes of failure detected during the pull off test.

**Table 6:** Pull off test results at 28 days.

Specimen designation	Bond strength (MPa)	Failure location
SSA-28	1.22 ± 0.08	Interface
SSE-28	1.15 ± 0.09	Interface
SMA-28	1.80 ± 0.19*	Substrate
SME-28	1.29 ± 0.09	Interface
FSSA-28	1.11 ± 0.15	Interface
FSSE-28	0.94 ± 0.15	Interface
FSMA-28	1.25 ± 0.10	Interface
FSME-28	1.22 ± 0.13	Interface
SM-28	1.41 ± 0.10	Interface
CM-28	1.25 ± 0.09*	Substrate
CMA-28	1.25 ± 0.07*	Substrate

* Shear stress because the failure location is in the substrate layer not the interface.

** Bond strength values represent mean ± standard deviation for three specimens.

Failure modes were classified based on visual inspection of fracture surfaces and crack propagation patterns recorded during testing.

– **Interface failure (pull-off)** was defined as debonding occurring entirely at the repair–substrate interface.
–**Substrate failure** was noted when the fracture propagated within the substrate concrete rather than along the bond line.

#### Pull off test parametric study

This study was done to evaluate the effect of each parameter included in the pull off test on the tensile bond strength between GPC as a repair layer and Portland cement concrete as a substrate layer.

As a general finding, it was found that the same trends observed during the analysis of the parametric study of the slant shear test were also observed in the parametric study of the pull off test, but with different ratios between the compared elements.

##### Effect of bonding agent

**Fig. 22:**
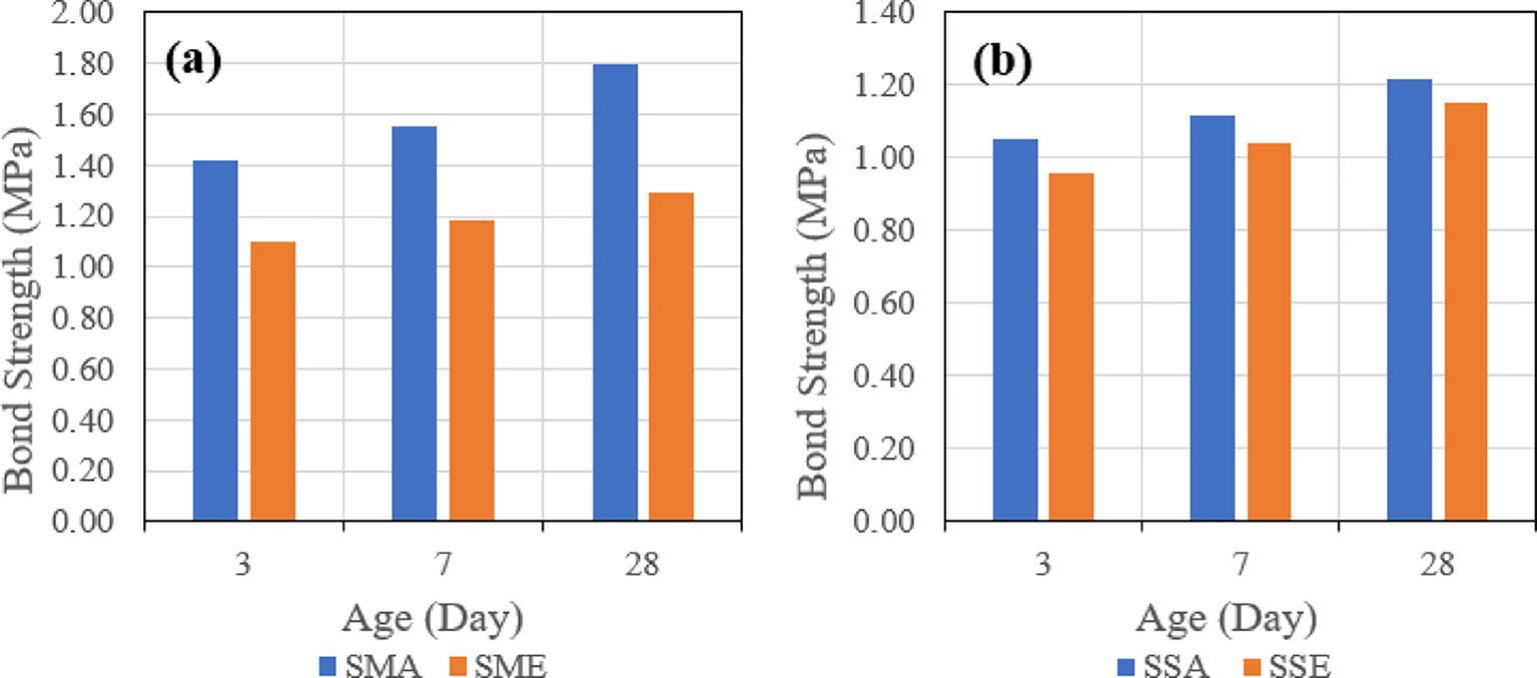
Effect of bonding agent on pull-off bond strength for slag-based geopolymer concrete (SGC) using (**a**) manual roughening and (**b**) sandblasting roughening at 28 days.

The first parameter evaluated is the effect of the bonding agent on the tensile bond strength. Figure 22 indicates the effect of used bonding agents when the same mix is used, which is (SGC), and the same roughening technique, which is the manual roughening in (a) and sandblasting roughening in (b).

**Fig. 23:**
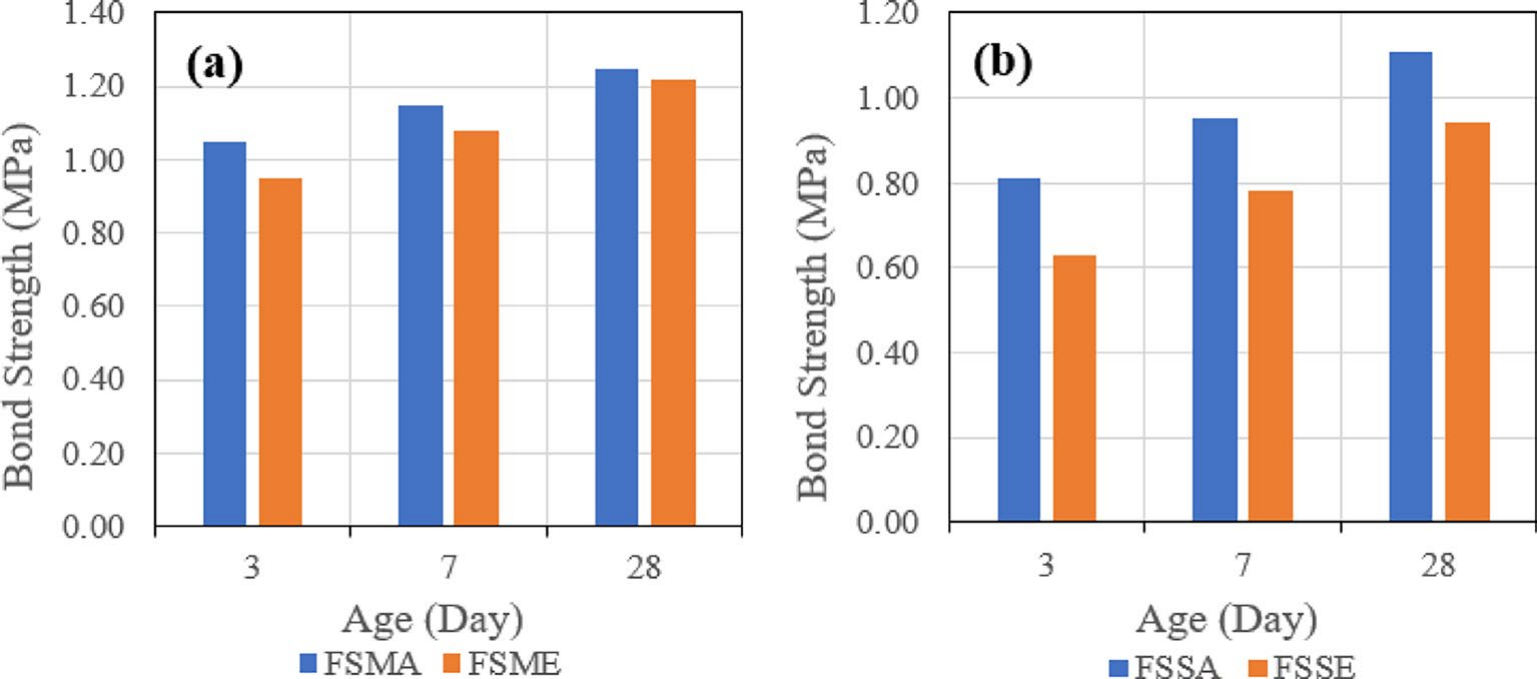
Effect of bonding agent on pull-off bond strength for fly ash–slag–based geopolymer concrete (FSGC) using (**a**) manual roughening and (**b**) sandblasting roughening at 28 days.

Figure 23 also represents the effect of used bonding agents when the same mix is used, which is (FSGC), and the same roughening technique, which is the manual roughening in (a) and sandblasting roughening in (b).

It was observed that in the two mixes, SGC and FSGC, the inclusion of acrylic bonding agent gave tensile bond strength greater than epoxy-based bonding agent. Also, the influence of the bonding agent is clear in the case of the SGC mix, as the difference between the two roughening techniques is higher than FSGC.

##### Effect of surface roughness

**Fig. 24:**
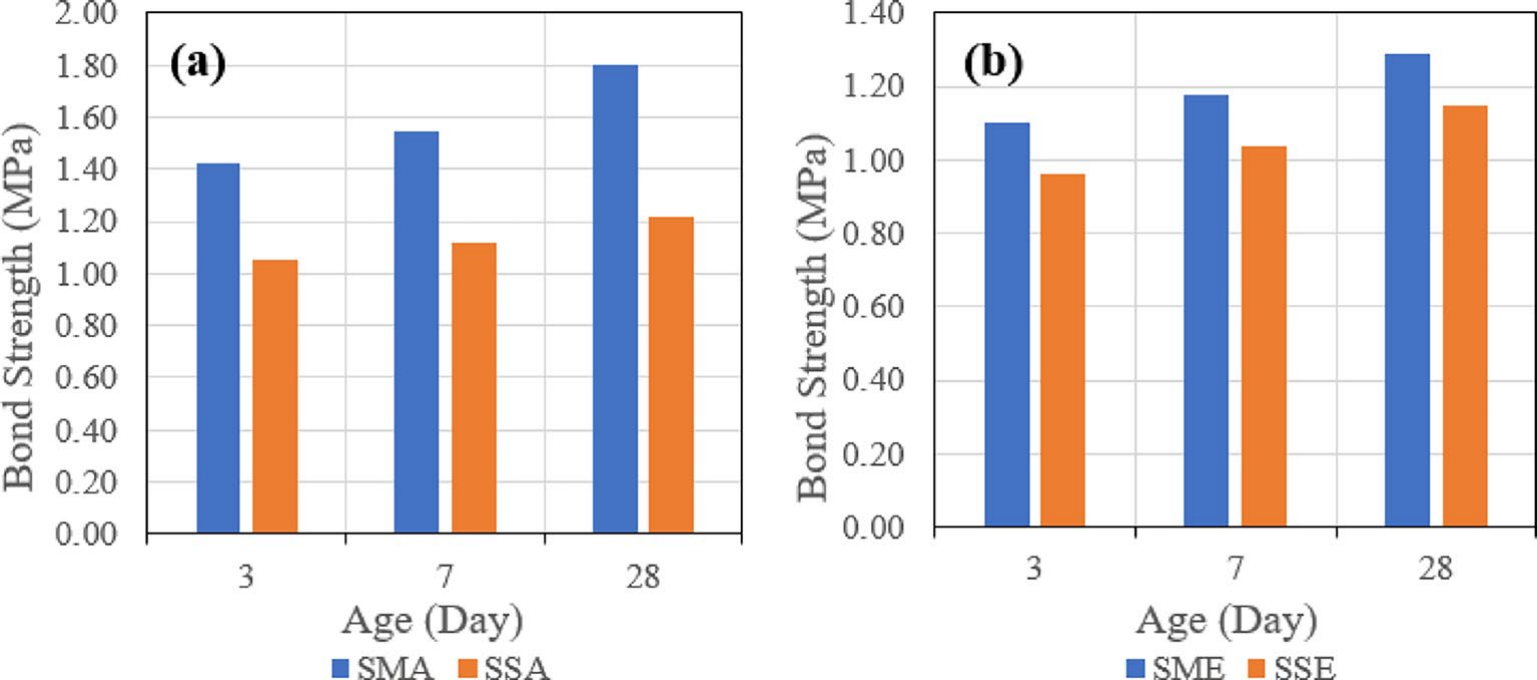
Effect of surface roughness (manual vs. sandblasting) on pull-off bond strength for slag-based geopolymer concrete (SGC) using (**a**) acrylic bonding agent and (**b**) epoxy bonding agent at 28 days.

The second evaluated parameter is the effect of surface roughness on the tensile bond strength. Figure 24 indicates the effect of the two types of surface roughness when the same mix is used, which is (SGC) and the same bonding agent, which is the acrylic bonding agent in (a) and the epoxy bonding agent in (b).

**Fig. 25:**
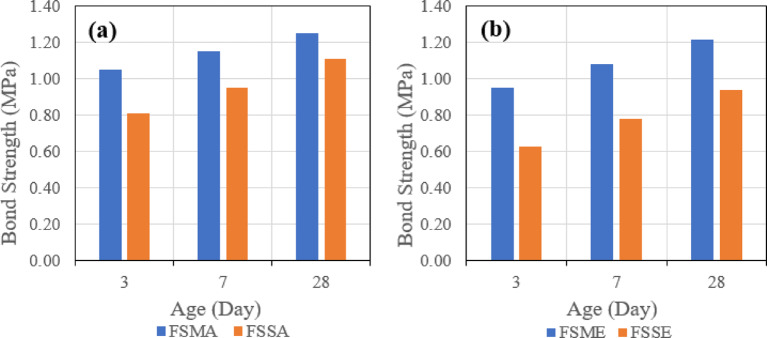
Effect of surface roughness (manual vs. sandblasting) on pull-off bond strength for fly ash–slag–based geopolymer concrete (FSGC) using (**a**) acrylic bonding agent and (**b**) epoxy bonding agent at 28 days.

Figure 25 also represents the effect of used surface roughness when the same mix is used, which is (FSGC), and the same bonding agent, which is the acrylic bonding agent in (a) and the epoxy bonding agent in (b).

It was observed that in the two mixes, SGC and FSGC, the usage of the manual roughening technique gave higher tensile bond strength between GPC and PCC than the sandblasting roughening technique.

##### Effect of binder

**Fig. 26:**
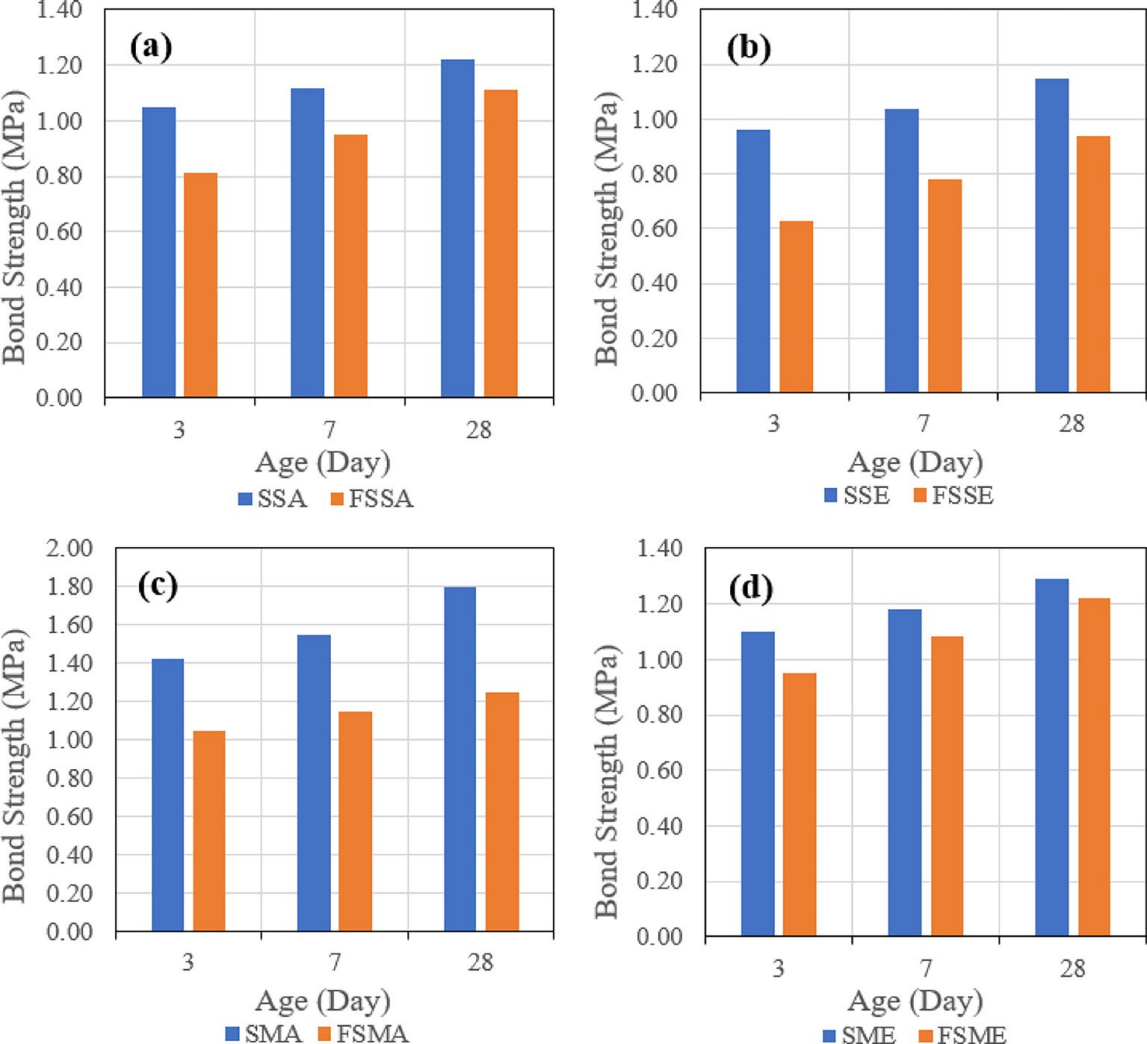
Effect of binder type (slag-based vs. fly ash–slag–based geopolymer concrete) on pull-off bond strength under combined conditions: (**a**) sandblasting + acrylic bonding agent, (**b**) sandblasting + epoxy bonding agent, (**c**) manual + acrylic bonding agent, and (**d**) manual + epoxy bonding agent at 28 days.

The third evaluated parameter is the effect of different binders on the tensile bond strength. Figure 26 indicates the effect of the two types of binders when the same roughening technique and the same bonding agent are used, which might be one of four combinations, which are Sandblasting + Acrylic (a), Sandblasting + Epoxy (b), Manual + Acrylic (c), and Manual + Epoxy (d).

It was observed that in the four combinations, the usage of the mix (SGC), which contains 100% slag, gave higher tensile bond strength. These results align with recent experimental findings^19^.

The superior bond strength of 100% slag-based GPC can be attributed not only to mechanical interlocking but also to chemical mechanisms at the interface. The high calcium content in slag leads to the formation of additional C-(A)-S-H type gels, which enhance the chemical adhesion with the hydrated phases of the PCC substrate. This contributes to a denser and more cohesive interfacial transition zone (ITZ). Similar observations were reported by^20^, where calcium-rich geopolymer mortars exhibited stronger bonding with OPC substrates due to improved chemical compatibility and reduced porosity at the interface.

It is important to note that the elastic modulus of GPC typically differs from that of Portland cement concrete, depending on binder composition and curing conditions. This mismatch in stiffness may influence stress distribution at the interface under load, leading to localized stress concentrations and affecting crack initiation and propagation. While this study did not include direct modulus measurements, previous studies report that high-slag GPC often exhibits a comparable or slightly lower modulus than PCC. Therefore, modulus mismatch could partly explain the variation in failure modes observed (sliding vs. splitting). Future studies should incorporate elastic modulus measurements and interface modeling to better understand the mechanical interaction between the two layers.

The scalability of these results to full-scale repairs must be carefully considered. While the specimens in this study were laboratory-scale, the observed bond behavior provides useful insights for practical applications. In real structures, additional factors such as structural restraint, load history, shrinkage compatibility, and environmental exposure can influence interface performance. The trends observed in this experimental program are likely transferable to practical retrofitting applications, provided that repair design adequately accounts for differential shrinkage, load histories, and environmental exposures.

## Conclusions

This study experimentally investigated the bond behavior between GPC repair materials and PCC substrates, considering the influence of surface roughening techniques (manual vs. sandblasting), bonding agents (acrylic vs. epoxy), and binder types (slag-based vs. fly ash–slag blends). Bond performance was evaluated using slant shear and pull-off tests, supported by surface roughness characterization and failure mode analysis. The following conclusions are drawn from the test results:

1. Surface preparation significantly influenced bond performance. Manual roughening increased surface roughness by approximately 60–80% compared to sandblasting, resulting in up to 50% higher bond strength in both slant shear and pull‑off tests.
2. Using a bonding agent achieved higher bond strength between GPC and Portland cement concrete than specimens with no bonding agent at the interface surface.
3. Binder composition affected bond behavior. GPC with 100% slag achieved the highest bond strengths, reaching 27.5 MPa in slant shear and 1.8 MPa in pull‑off tests, outperforming Portland cement concrete by about 25–35% under similar interface conditions.
4. Specimens with manual roughening technique and acrylic bonding agent achieved splitting mode failure, indicating the good bond between GPC and Portland cement concrete.
5. GPC contains 100% slag, achieved higher bond strength than Portland cement concrete as a repair layer at the same interface conditions while GPC contains 60% slag and 40% fly ash, achieved lower bond strength than Portland cement concrete as a repair layer at the same interface conditions.

Properly designed slag-based geopolymer repair systems, especially when combined with manual surface roughening and acrylic bonding agents, can deliver substantially improved bond performance compared to conventional PCC repairs, highlighting their potential as durable and high-performance solutions for structural retrofitting.

## Further research

Although this study assessed short-term bond behavior, the long-term durability of the geopolymer–Portland cement concrete interface remains critical for practical applications. Thermal cycling can induce differential expansion and contraction between the repair and substrate layers, potentially weakening the interface over time. Moisture ingress may cause microcracking and alter the geopolymer matrix chemistry, while exposure to aggressive agents (chlorides, sulfates, acids) can deteriorate both the interface and the surrounding concrete. While this study focused on short-term bond strength at ambient conditions, future research should investigate the long-term durability of the geopolymer–Portland cement concrete interface under environmental stressors. This includes:

- Thermal cycling to evaluate the impact of repeated heating–cooling on interfacial stability;
- Wet–dry and freeze–thaw cycles to simulate moisture-induced stresses and frost damage;
- Chemical exposure tests (chlorides, sulfates, acids) to assess interface degradation in aggressive environments.

Additionally, advanced characterization techniques such as micro-CT scanning and nanoindentation could provide deeper insights into interfacial microstructural changes over time. These studies will help establish the long-term viability of GPC for structural retrofitting applications.

## Data Availability

The datasets generated and/or analysed during the current study are available from the corresponding author on reasonable request.
